# *Plasmodium falciparum*-Specific Memory B-Cell and Antibody Responses Are Associated With Immunity in Children Living in an Endemic Area of Kenya

**DOI:** 10.3389/fimmu.2022.799306

**Published:** 2022-03-09

**Authors:** Peter Jahnmatz, Diana Nyabundi, Christopher Sundling, Linnea Widman, Jedidah Mwacharo, Jennifer Musyoki, Edward Otieno, Niklas Ahlborg, Philip Bejon, Francis M. Ndungu, Anna Färnert

**Affiliations:** ^1^ Division of Infectious Diseases, Department of Medicine Solna and Center for Molecular Medicine, Karolinska Institutet, Stockholm, Sweden; ^2^ Mabtech AB, Nacka Strand, Sweden; ^3^ KEMRI - Wellcome Research Programme/Centre for Geographical Medicine Research (Coast), Kilifi, Kenya; ^4^ Department of Infectious Diseases, Karolinska University Hospital, Stockholm, Sweden; ^5^ Division of Biostatistics, Institute of Environmental Medicine, Karolinska Institutet, Stockholm, Sweden; ^6^ Department of Molecular Biosciences, The Wenner-Gren Institute, Stockholm University, Stockholm, Sweden; ^7^ Centre for Tropical Medicine and Global Health, University of Oxford, Oxford, United Kingdom

**Keywords:** *P.falciparum* malaria, recombinant antigens, memory B-cells, antibodies, FluoroSpot

## Abstract

Identifying the mechanism of naturally acquired immunity against *Plasmodium falciparum* malaria could contribute to the design of effective malaria vaccines. Using a recently developed multiplexed FluoroSpot assay, we assessed cross-sectional pre-existing memory B-cells (MBCs) and antibody responses against six well known *P. falciparum* antigens (MSP-1_19_, MSP-2 (3D7), MSP-2 (FC27), MSP-3, AMA-1 and CSP) and measured their associations with previous infections and time to clinical malaria in the ensuing malaria season in Kenyan children. These children were under active weekly surveillance for malaria as part of a long-term longitudinal malaria immunology cohort study, where they are recruited from birth. After performing Cox regression analysis, we found that children with a breadth of three or more antigen-specific MBC or antibody responses at the baseline had a reduced risk for malaria in the ensuing *P. falciparum* transmission season. Specifically, MBC responses against AMA-1, MSP-2 (3D7) and MSP-3, as well as antibody responses to MSP-2 (3D7) and MSP-3 were prospectively associated with a reduced risk for malaria. The magnitude or breadth of MBC responses were however not correlated with the cumulative number of malaria episodes since birth. We conclude that increased breadth for merozoite antigen-specific MBC and antibody responses is associated with protection against malaria.

## Introduction

*Plasmodium falciparum* malaria is a leading cause of death in Sub-Saharan Africa, especially in children. Globally, over 241 million malaria cases and 627,000 related deaths were reported in 2020 (1, 2). Children living in high endemic areas are at particular risk of life threatening malaria before gradually acquiring immunity, which requires repeated exposure (3). The lead malaria vaccine, RTS,S, is insufficiently protective and a more effective vaccine is needed (4, 5). In order to develop such a vaccine, a better understanding of the biological processes leading to natural acquired immunity is needed.

Protection against the most severe forms of malaria is achieved more rapidly than against uncomplicated malaria, with immunity against uncomplicated malaria developing gradually after repeated parasite exposures in children living in endemic areas (6, 7). However, this clinical immunity has been shown to decline in the absence of continuous exposure, resulting in a loss of protection against re-infections (8).

Antibodies specific for different parasite antigens have been identified as important components of naturally acquired immunity (9–11), although the mechanisms of this antibody-based immunity are not completely understood. Antibodies have been found to bind to the surface antigens on the parasite, thereby blocking its invasion of hepatocytes and red blood cells, activating complement-associated lysis of merozoites, inhibiting parasite egress from schizonts, and mediating parasite opsonophagocytosis *in vitro* (12–17). Plasma levels of antibodies to various *P. falciparum* antigens have been associated with protection, and are also used to assess exposure and changing transmission patterns, in immunoepidemiological studies (18–21). However, antibody responses against *P. falciparum* antigens have been found to be short-lived (19), especially in young children living in endemic areas (22), and may also be highly transient during malaria seasons (8, 19, 23). Development of naturally acquired immunity against *P. falciparum* is further constrained by the extensive genetic diversity, including antigenic variation and polymorphisms displayed by many of the parasite antigens (24, 25).

As a complement to studies on antibody responses, increased focus has been directed towards circulating antigen-specific memory B-cells (MBCs). Although MBCs by themselves are unlikely to neutralize infectious agents, they are critical for maintaining anamnestic antibody driven immunity, whereby they rapidly proliferate and differentiate into antibody secreting cells in response to antigen re-stimulation (26). In humans, these quiescent MBCs have been shown to be located in both secondary lymphoid organs and in blood (27).

Circulating malaria specific MBCs can be long-lived even in the absence of re-exposure to parasites, whilst antibodies may decay to below detectable levels over time (28, 29). Thus, studies investigating the role of antigen-specific antibody responses in immunity could provide complementary information by including parallel analysis of antigen-specific MBCs. This has not always been possible for a majority of immunoepidemiological studies as the methods for quantifying antigen-specific MBCs are highly demanding due to the large volumes of blood required for peripheral blood mononuclear cell (PBMC) isolation. Moreover, the methods involved have been laborious as only one antigen could be tested at a time in ELISpot assays. These limitations are especially apparent in studies involving small volumes of blood from young children in malaria endemic areas, who also happen to be the most important group to study as they are the most affected by malaria. To solve this problem, we recently developed and validated a novel reversed B-cell FluoroSpot assay with the capacity to simultaneously detect MBCs against multiple parasite antigens from the same sample in the same well (30).

Here, we have used this multiplexed FluoroSpot assay to measure the frequencies of MBCs specific for six well known *P. falciparum* antigens [merozoite surface protein 1 (19) (MSP-1_19_), MSP-2 (3D7), MSP-2 (FC27), MSP-3, apical membrane antigen 1 (AMA-1) and circumsporozoite protein (CSP)] in children living in an endemic region of Kenya. Circulating antibody responses to these antigens have previously been identified as possible markers of immunity against malaria (25, 31–34). We compared antigen-specific MBCs against their cognate circulating antibodies in their ability to predict immunity to malaria, as well as previous exposure.

## Materials And Methods

### Study Area

The study cohort involved children from two regions, Junju and Ngerenya, within Kilifi County, Kenya. The regions are located within 20 km of each other and are separated by the Kilifi Indian Ocean Creek. However, they experience different levels of malaria transmission, most of which occurs during two distinct annual rainy seasons: June-August, and November-December (35). While *P. falciparum* transmission in Ngerenya had declined to extremely low levels by the time of this study, malaria transmission in Junju remained relatively stable with a prevalence of asymptomatic *P. falciparum* infections at 25-30% among children (36).

### Study Population

Children in the Ngerenya and Junju cohorts have been under active weekly surveillance for malaria since 1998 and 2005, respectively. In both cases, children are recruited at birth and remain under surveillance until their 15^th^ birthday. The weekly visit by field workers involves testing for malaria in cases where the child is febrile. Treatment is given based on a *P. falciparum* rapid diagnostic test (RDT; CareStart™ Malaria Test, AccessBio, NJ, USA) and blood smears for microscopy. All children with RDT positive tests are treated with artemether/lumefantrine (Coartem^®^, Novartis, Basel, Switzerland). All positive RDT results are confirmed by microscopy. For the purpose of this study, the definition of a clinical malaria episode was set as having an axillary body temperature of ≥37.5°C combined with a *P. falciparum* parasite density of ≥2500 parasites per microliter of blood (37). Active surveillance is still ongoing in Junju but was discontinued in favor of passive surveillance in 2015 for Ngerenya children due to extremely low levels of malaria transmission (38). An annual cross-sectional survey is held in March in both cohorts, where venous blood samples, anthropometric and parasitological data are collected for immunology studies. Blood samples are processed into PBMC and plasma, which are stored in liquid nitrogen and -80°C, respectively, until use.

**Figure 1:**
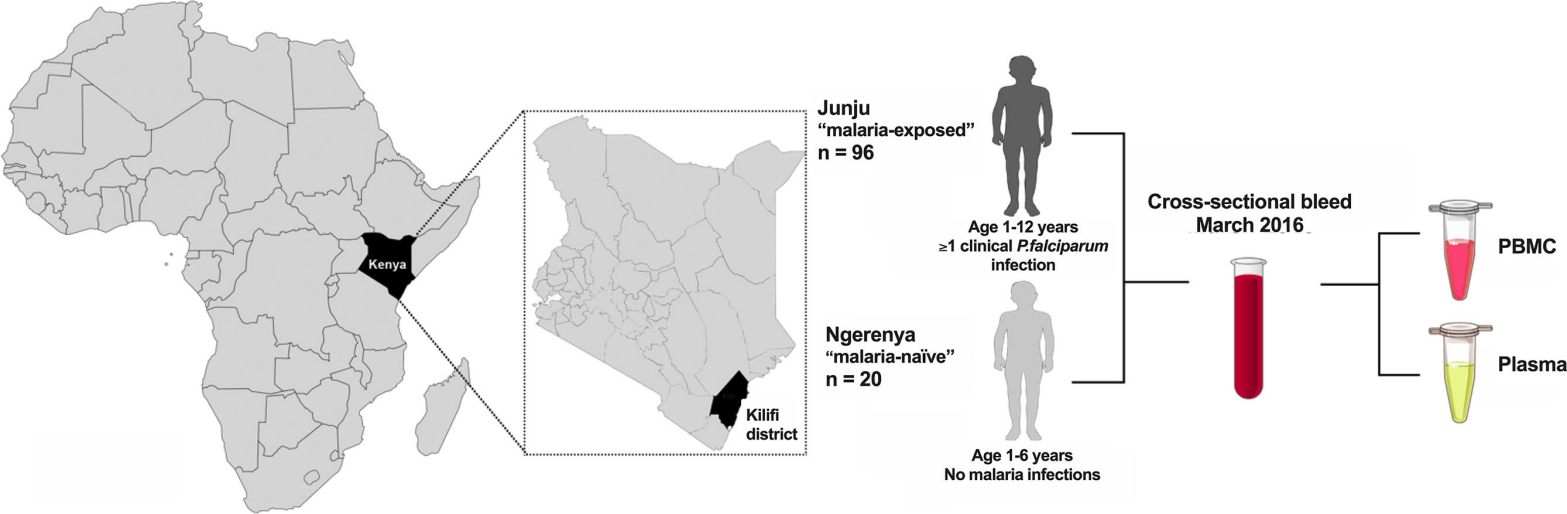
Schematics of study. Study participants were children from the two regions Junju and Ngerenya in the Kilifi district of Eastern Kenya. The Junju children (n=96) all had at least one episode of clinical malaria while Ngerenya childen (n=20) were malaria naïve controls. Peripheral blood mononuclear cells (PBMC) and plasma were isolated during the cross-sectional survey of March 2016.

For this study, we used samples collected in the cross-sectional survey of March 2016 (**Figure 1**). In Junju, children (1-12 years) were still under active weekly surveillance for fever and detection of malaria parasites through home visits (compliance 94.9%, range 33.3-100%) and annual cross-sectional surveys (compliance 98.1%, range 88.7-100%). In contrast, the Ngerenya children (1-6 years) included in this study as malaria naïve controls for antigen-specific immune responses were born after the complete control of malaria in the area. We also confirmed that the Ngerenya children were all negative for asymptomatic *P. falciparum* infections by both PCR and microscopy from all the previous cross-sectional surveys, and that they had no recorded malaria episodes since birth, as determined from the passive surveillance.

### Sample Collection and Parasite Detection

In March 2016, prior to the main rainy season, 5 ml of venous blood were drawn from each child by venesection into heparinized tubes. PBMC and plasma samples were processed and stored in liquid nitrogen and -80°C, respectively, until use. Examination for malaria parasites was performed at all annual surveys by microscopy with thick and thin blood smear stained with 10% Giemsa and 1x1000 magnification (39, 40). In addition, samples were examined for malaria parasites by *Plasmodium* species-specific real-time PCR as previously described (41).

### Antigen Preparation

*P. falciparum* merozoite antigens MSP-1_19_, MSP-2 (3D7), MSP-2 (FC27), MSP-3 and AMA-1 were expressed recombinantly in a mammalian expression system for use in the FluoroSpot and peptide tag ELISA assays, respectively, as described elsewhere (30). CSP was expressed, based on Uniprot accession number P19597, using the same method as the merozoite antigens. The merozoite antigens were tagged with the following peptide tags as described previously (42): MSP-1_19_ with BAM; MSP-2 (3D7) with GAL; MSP-2 (FC27) with Twin-Strep-tag^®^ (IBA LifeSciences, Goettingen, Germany); MSP-3 with WASP, AMA-1 with Twin-Strep-tag^®^; and the newly expressed CSP was tagged with the peptide tag WASP.

### Reversed B-Cell FluoroSpot Assay

**Figure 2:**
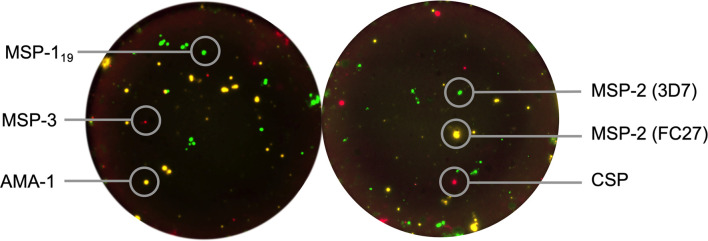
Reversed B-cell FluoroSpot readout with *Plasmodium falciparum* antigens. Computerized overlay of images from a FluoroSpot readout with a donor responding to all parasite antigens. To the left, green spots represent spot-forming units (SFU) from memory B cells (MBC) specific against *P. falciparum* MSP-1_19_, yellow; AMA-1 and red; MSP-3. To the right, green spots represent SFU from MBC specific against MSP-2 (3D7), yellow; MSP-2 (FC27) and red; CSP.

The reversed B-cell FluoroSpot was multiplexed with three antigens simultaneously in two separate wells combining MSP-1_19,_ MSP-3 and AMA-1 in one well, and MSP-2 (3D7), MSP-2 (FC27) and CSP in another (**Figure 2**). Briefly, plates (Merck Millipore, Burlington, MA, USA) were precoated with a monoclonal antibody (mAb) anti-human IgG (mAb MT91/145 from Mabtech) and blocked in culture medium. Thereafter, 250,000 cells that previously had been cultured for 5 days at 37°C and 5% CO_2_ in the presence of 1 μg/mL R848 and 10 ng/ml of recombinant IL-2 (both from Mabtech), were added to the wells used for the multiplex MBC antigen-specific analysis; and 25,000 cells to wells used for MBC total IgG analysis. Cells were then cultivated in these wells for 20-24 hours in 37°C and 5% CO_2_. Plates were washed, followed by addition of combinations of supernatants containing the respective tagged parasite antigens diluted in PBS and 0.1% BSA (Sigma-Aldrich, Saint Louis, MO, USA) (PBS/BSA) and incubated at room temperature (RT) for 1 hr. Irrelevant control antigens (cow-, horse-, dog- or woodchuck IFN-γ tagged with the same peptide tags as the parasite antigens) were added in separate wells. After another wash, fluorophore-conjugated anti-tag antibodies or fluorophore-conjugated Strep-Tactin^®^XT (IBA LifeSciences) were added to wells an incubated for 1 hr at RT in PBS/BSA supplemented with filtered human plasma diluted 1:100. For the total IgG analysis, a biotinylated anti-human IgG (mAb MT78/145-biotin from Mabtech) followed by fluorescently conjugated streptavidin (SA) were added instead. After a last wash, Fluorescent enhancer (Mabtech) was added to the plates and incubated for 10 minutes at RT before plate underdrains were removed, and plates were dried in the dark at RT. The FluoroSpot plates were analysed using the Mabtech IRIS™ reader system equipped with Apex™ software version 1.1.7. Definition of assay disqualification was applied to samples with <1000 total IgG spot forming units (SFU) per 250,000 cells. Magnitudes of MBC responses were expressed as proportions of antigen-specific IgG spots per total IgG spots (%MBC/total IgG) and SFU per 10^6^ PBMCs after subtracting reactivity to negative control antigens (i.e., the respective peptide tags used in the expression and purification of the *P. falciparum* recombinant proteins). The frequency of MBCs to the expression tag control antigen was low for all antigens (median 3, range 0-24 SFU/10^6^ PBMCs, **Supplementary Figure 1**). Threshold for MBC positivity was then set to ≥20 SFU per 10^6^ PBMCs corresponding to ≥5 SFU per well with 250,000 PBMCs to account for artefact spots in the assay.

### Peptide Tag-Based ELISA

Plasma samples were analysed by ELISA for detection of circulating IgG antibodies against *P. falciparum* MSP-1_19_, MSP-2 (3D7), MSP-2 (FC27), MSP-3, AMA-1 and CSP as well as a negative control antigen (horse IFN-γ) as previously described (30). Briefly, ELISA plates were coated with Strep-Tactin^®^XT (IBA LifeScience) at a concentration of 1 μg/mL in PBS and incubated overnight. The following day, additional binding sites were blocked by the addition of 100 µL of PBS supplemented with 0.1% Tween 20, 0.1% BSA (both from Sigma-Aldrich) (incubation buffer), and incubated for 1 hr at RT. Plates were then washed in PBS supplemented with 0.1% Tween 20 (Sigma) followed by addition of Twin-Strep^®^ tagged variants of the *P. falciparum* antigen diluted 1:5 in incubation buffer. Plates were then incubated for 1 hr at RT. After another wash, human plasma samples diluted to 1:2000 in incubation buffer was added to plates and incubated for 1 hr in RT. Plasma antibodies having bound the respective antigen were detected by addition of horse-radish peroxidase conjugated anti-human IgG mAb (Mabtech AB) at a concentration of 0.5 µg/mL followed by subsequent addition of TMB substrate to develop signal. The reaction was stopped by addition of ELISA Stop solution (Mabtech AB) and the plates were read in a Synergy HTX Absorbance reader and analysed using Gen5 software (both from Bio Tek, Winooski, VT, USA). Mean OD values are presented as antibody levels.

### Statistical Analyses

Statistical analyses were performed using STATA MP version (16.0), and GraphPad Prism (version 8.3) (GraphPad Software, La Jolla, CA). Mann-Whitney test was used to compare IgG spots (%MBC/total IgG), spot forming units (SFU)/10^6^ PBMCs and antibody levels between two age groups (1-6 and 7-12 years old) within the children in Junju (defined as malaria-exposed) and children in Ngerenya (defined as malaria-naïve) aged 1-6 years. Spearman correlation was used to determine the association between logarithmically transformed MBC and antibody responses, as well as to determine the association of MBC and antibody responses with age, number of clinical malaria episodes since birth and parasite density at baseline in separate multivariate analysis. A Cox-regression model was used to investigate the risk of subsequent clinical malaria after baseline (date of sample collection in March 2016) until one year later, and similarly for time since last malaria episode until baseline. The models were performed by either subgrouping individuals considered positive or negative for MBC and antibody responses against an antigen or performed with number of SFU/10^6^ PBMC. In addition, the risk analyses were also performed with the levels of MBC responses as continuous values. Proportional hazards were tested using Schoenfeld’s residuals. The *p*-values below 0.05 and hazard ratio (HR) with 95% CI not crossing 1 were considered significant.

## Results

### Heterogeneity in the Magnitude of MBC Responses to Different *P. falciparum* Antigens

Among the 316 children participating the Junju-cohort March 2016 cross sectional survey, 96 children, aged 1-12 years were selected for the analyses of antigen-specific antibody and MBC responses. Children in the Juju cohort were selected based on their prior exposure status: having a history of at least one clinical episode of *P. falciparum* malaria since birth. None of them had symptoms of clinical malaria either at the time, or within 15 days before and after sampling. However, 16 of the 96 (16%) children had asymptomatic *P. falciparum* infection detected by PCR at sampling. In addition, 20 children from the Ngerenya cohort, aged 1-6 years, who had no recorded malaria episode during their lifetime, or detection of asymptomatic parasites in either the current, or in any of the preceding annual surveys (since birth) were included as a malaria naïve comparison group (**Table 1**). Hereafter, children from Junju are referred to as “malaria-exposed” and children from Ngerenya as “malaria-naïve”.

**Table 1:** Characteristics of study participants from the Junju and Ngerenya cohorts.

	Junju (malaria-exposed)	Ngerenya (malaria-naïve)
Number of children	96	20
Female, n (%)	46 (48)	8 (40)
Age, years, median (range)	6 (1-12)	4 (1-6)
*P. falciparum* parasites detected by PCR at sample collection, n (%)	16 (18)	0
Number of clinical malaria episodes since birth, median (range)	8 (1-28)	0
Days since last clinical malaria episode before sample collection, median (range)	94 (34-2764)	0 (0-0)
Participants with a clinical *P. falciparum* infection during follow up, n (%)	83 (86)	0

**Figure 3:**
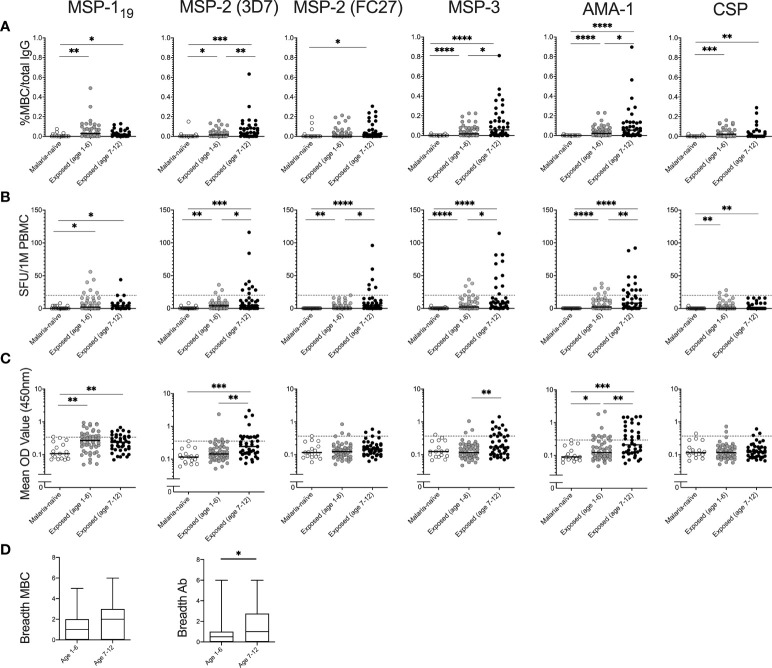
Magnitude of memory B-cell (MBC) and antibody responses to *Plasmodium falciparum* antigens in children with and without history of parasite exposure. Magnitude of MBC responses to parasite antigens measured by B-cell FluoroSpot in exposed children of different age groups (Junju) as well as malaria naïve controls (Ngerenya) displayed as **(A)** proportion of MBC per total IgG producing cells, or **(B)** Spot-forming units (SFU) per million peripheral blood mononuclear cells (PBMC). Dotted line indicate threshold for positivity. **(C)** Antibody responses to *P. falciparum* antigens measured by ELISA. Dotted line indicate threshold for positivity. **(D)** Breadth of MBC and antibody responses of malaria-exposed children of different age groups as measured by FluoroSpot and ELISA respectively. The breadth of the response was defined as the number of antigens against which an individual had reactivity above threshold. Differences between groups were evaluated by Mann-Whitney test. *p < 0.05, **p < 0.01, ***p < 0.001, ****p < 0.0001.

The magnitude of the MBC responses differed between antigens and between the two comparison groups (**Figure 3A**). Frequencies of antigen-specific MBCs among malaria-exposed children exceeded the malaria-naïve controls for all antigens except for MSP-2 (FC27) and CSP, where these responses were at background levels. In the malaria-exposed children, median frequency of MBCs specific to MSP-1_19_ was 4 SFU/10^6^ PBMCs (range 0-192), median 4 (range 0-240) for MSP-2 (3D7), median 0 (range 0-96) for MSP-2 (FC27), median 4 (range 0-204) for MSP-3, median 4 (range 0-344) for AMA-1 and median 0 (range 0-28) for CSP (**Figure 3A**).

Cross-reactivity (i.e., co-positioned spots in the multiplex FluoroSpot assay) was not observed between the two allelic forms of MSP-2 (3D7 and FC27), when assessing spots counted using different wavelength-specific filters. Data from six malaria-exposed children were excluded from the analysis due to assay disqualification i.e., <1000 total IgG spot-forming units (SFU) per 250,000 cells in the FluoroSpot assay. Furthermore, MSP-1_19_ and CSP MBC data from 28 children were excluded from the analysis owing to technical challenges and could not be repeated due to limitations in PBMC numbers.

### *P. falciparum*-Specific MBC and Antibody Levels Increase With Age

The proportions of malaria-exposed children qualifying as MBC positive were 19/90 (21%) for AMA-1, 17/90 (19%) for MSP-3, 13/90 (14%) for MSP-2 (3D7), 7/90 (8%) for MSP-2 (FC27), 10/62 (16%) for MSP-1_19_, and 3/62 (5%) for CSP. None of the responding children had MBC responses to all antigens tested, 3 children (3.3%) responded to four antigens, 9 children (10%) responded to three antigens, 7 children responded to two antigens (7.8%), 16 children (17.7%) responded to one antigen, and 55 children (61.1%) were negative to all the antigens. All the malaria-naïve children were negative against all the antigens tested.

The median frequencies of MBCs for MSP-2 (3D7), MSP-3 and AMA-1 were higher in children aged 7-12 years compared to 1–6-year old’s (Mann-Whitney test *p*= 0.007, 0.022 and 0.022, respectively) (**Figure 3B**). Similarly, frequencies of MBCs for all antigens except for CSP were positively correlated with age (Spearman correlation coefficient r_s_ -0.273 *p*=0.031 for MSP-1_19_, r_s_ 0.318 *p*=0.002 for MSP-2 (3D7), r_s_ 0.298 *p*=0.004 for MSP-2 (FC27), r_s_ 0.213 *p*=0.044 for MSP-3 and r_s_ 0.284 *p*=0.006 for AMA-1). Furthermore, the breadth of MBC responses was also weakly correlated with age (r_s_ 0.258 *p*=0.014) (**Table 2**).

**Table 2:** Correlation of MBC and antibody responses with age, cumulative number of clinical infections since birth and parasite positivity at sample collection.

	Age		Number of clinical *P.falciparum* episodes since birth to sample collection		Parasite positivity at sample collection		Days since last previous clinical *P.falciparum* infection	
	Coefficient	p	Coefficient	p	Coefficient	p	Coefficient	p
MBC MSP-1_19_	**-0.273**	**0.031**	-0.194	0.131	-0.148	0.250	0.071	0.071
MSP-2 (3D7)	**0.318**	**0.002**	0.045	0.674	**0.274**	**0.009**	-0.126	0.235
MSP-2 (FC27)	**0.298**	**0.004**	0.043	0.688	**0.289**	**0.006**	-0.161	0.129
MSP-3	**0.213**	**0.044**	-0.125	0.239	**0.300**	**0.004**	-0.179	0.089
AMA-1	**0.284**	**0.006**	-0.064	0.550	**0.459**	**<0.001**	-0.087	0.411
CSP	-0.036	0.779	0.001	0.963	0.046	0.721	-0.045	0.726
Ab MSP-1_19_	-0.143	0.177	**-0.307**	**0.003**	0.027	0.796	0.001	0.993
MSP-2 (3D7)	**0.281**	**0.007**	-0.051	0.631	**0.342**	**<0.001**	-0.178	0.093
MSP-2 (FC27)	0.095	0.371	-0.147	0.166	0.144	0.176	-0.04	0.722
MSP-3	**0.212**	**0.045**	-0.149	0.161	**0.218**	**0.038**	-0.092	0.382
AMA-1	**0.208**	**0.049**	-0.030	0.779	**0.355**	**<0.001**	**-0.213**	**0.044**
CSP	0.043	0.688	-0.087	0.414	-0.038	0.718	0.006	0.955
Breadth^a^ MBC	**0.258**	**0.014**	-0.074	0.488	**0.359**	**<0.001**	-0.156	0.141
Ab	**0.248**	**0.018**	-0.131	0.216	**0.332**	**<0.001**	-0.171	0.108
Total IgG	0.124	0.239	0.036	0.737	0.053	0.689	0.117	0.273

Spearmans correlation coefficient indicating the association with immune responses and age, number of clinical episodes of malaria, parasite positivity by PCR at baseline.

a Breadth was defined as number of antigens (0-6) an individual had above threshold (20 spot-forming units for memory B cell responses or mean reactivity of malaria-naïve children + 2 SD). Values presented in bold represent a correlation with a p value below 0.05 and thereby considered to be statistically significant.

Median antibody OD levels were higher for MSP-2 (3D7) (Mann-Whitney test *p*=0.001), MSP-3 (Mann-Whitney test *p*=0.006) and for AMA-1 (Mann-Whitney test *p*=0.009) in children aged 7-12 compared to 1-6 year old’s among malaria-exposed children (**Figure 3C**). Antibody levels were positively correlated with age for MSP-2 (3D7) (Spearman correlation coefficient r_s_ 0.239, *p*=0.025) and MSP-3 (r_s_ 0.23, *p*=0.031) (**Table 2**). Breadth of antibody responses was also weakly correlated with age (r_s_ 0.244, *p*=0.023) (**Table 2**), and the median breadth of antibody responses was higher in children aged 7-12 years compared to 1-6 years (median 1 compared to 0.5, Mann-Whitney U-test *p*=0.011) (**Figure 3D**).

### Correlations Between Frequencies of Antigen-Specific MBCs and Antibody Levels

Frequencies of MBCs were positively but weakly correlated with antibody levels for MSP-2 (3D7) (Spearman correlation coefficient r_s_ 0.221 *p*=0.038), MSP-2 (FC27) (r_s_ 0.347 *p ≤* 0.001), MSP-3 (r_s_ 0.274 *p*=0.09) and AMA-1 (r_s_ 0.420 *p*= *p ≤* 0.001). However, this was not the case for MSP-1_19,_ and neither was it for CSP (**Supplementary Figure 2**).

### Correlation of the Breadth of MBC and Antibody Responses With Cumulative Number of Clinical Malaria Episodes Since Birth

The frequency of MBCs did not correlate with the cumulative number of clinical malaria episodes since birth for any of the antigens tested (**Table 2**). However, antibody levels against MSP-1_19_ were negatively correlated with the cumulative number of clinical malaria episodes since birth (r_s_ -0.307 *p*=0.003). The point estimates for antibody levels to the other antigens as well as their breadth showed a negative but non-significant correlation with the number of clinical episodes since birth (**Table 2**).

### Correlation of Antigen-Specific MBCs and Antibody Responses With the Time Since the Last Clinical Episode

Number of days since previous clinical episode of *P. falciparum* malaria was not correlated with the frequency of MBCs for any of the antigens tested. However, antibody levels against AMA-1 were weakly negatively correlated with number of days since malaria episode (r_s_ -0.213, *p*=0.044) (**Table 2**). Furthermore, the number of days since last clinical malaria episode was not correlated with breadth of neither frequency of MBCs nor antibody levels (**Table 2**).

### Correlation of Antigen-Specific MBC and Antibody Responses With Contemporaneous Asymptomatic *P. falciparum* Parasitemia

At the time of sample collection in March 2016, 16 (17.7%) of the 90 malaria-exposed children (5 aged 1-6 years, and 11 aged 7-12 years) with MBC data had asymptomatic *P. falciparum* parasitemia detected by microscopy and PCR. Parasite positivity at baseline was positively correlated with frequencies of MBCs and levels of antibodies against MSP-2, MSP-3 and AMA-1, as well as breadth of MBC responses (**Table 2**). Lastly, frequencies of MBCs of parasite positive children were higher in children aged 7-12 years compared to children aged 1-6 years (**Supplementary Figure 3**).

### Association of Breadth and Magnitude of Baseline Antigen-Specific MBC and Antibody Responses With the Prospective Risk of Clinical Malaria

**Figure 4:**
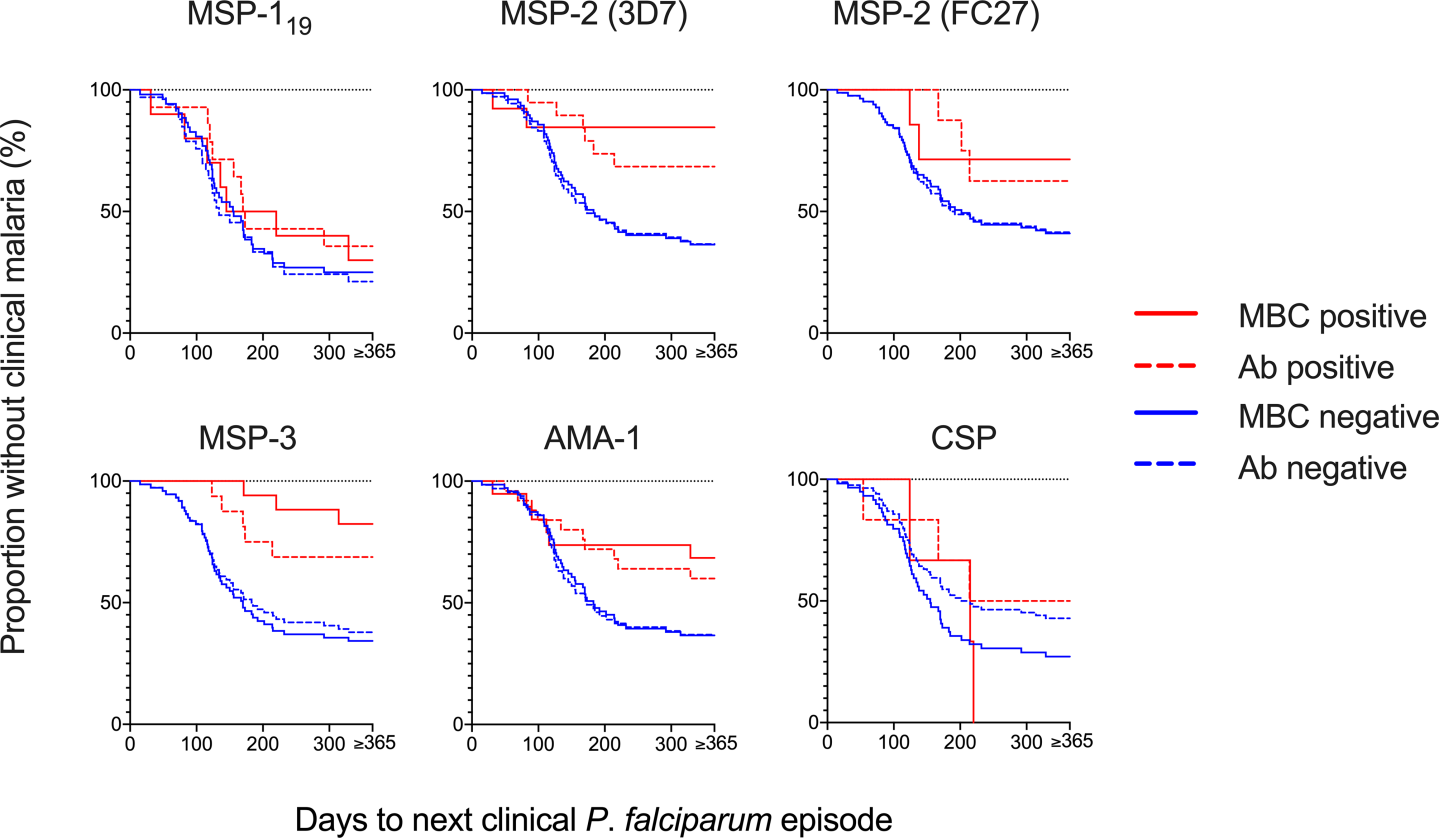
Risk of a clinical episode of malaria in relation to memory B-cell (MBC) and antibody positivity at baseline. The Kaplan-Meier curves present the proportion of malaria-exposed children (Junju) without a clinical episode, based on MBC positivity (solid line) and antibody positivity (dashed line) at baseline. The blue lines represent positive children and red lines the negative children, respectively. Positivity was defined as a reactivity above threshold. Unadjusted and adjusted Cox regression analyses of the risk of malaria are presented in **Table 3**.

Since the MBC and antibody levels were measured from samples collected in March, towards the end of a 4-month dry season when malaria transmission is very low, we assessed the prospective risk of subsequent clinical malaria associated with MBC and antibody positivity during the ensuing malaria transmission season starting from May. Time to a clinical episode of malaria from baseline in children with and without MBC and antibody responses, respectively, is visualized in **Figure 4**. The risk of malaria was further assessed in Cox regression models, in the first unadjusted models, the risk of malaria was reduced for individuals that were MBC positive for MSP-2-3D7 (HR 0.18, 95% CI 0.04-0.75, *p*=0.019), MSP-3 (HR 0.17, 95% CI 0.05-0.54, *p*=0.0.003) and AMA-1 (HR 0.41, 95% CI 0.17-0.97, *p*=0.044) (**Table 3**). Similarly, antibody positivity for MSP-2 (3D7) (HR 0.36, 95% CI 0.15-0.86, *p*=0.021) and MSP-3 (HR 0.36, 95% CI 0.14-0.92 *p*=0.033) were associated with a reduced risk of malaria (**Table 3**). After adjusting for age, the risk of malaria was reduced for individuals that were MBC positive for MSP-2 (3D7) (HR 0.17, 95% CI 0.04-0.73, *p*=0.017), and MSP-3 (HR 0.15, 95% CI 0.04-0.50, *p*=0.002). Also, after adjusting for parasite positivity the risk of malaria was only reduced for individuals that were MBC positive for MSP-3 (HR 0.21, 95% CI 0.06-0.69, *p*=0.01). Finally, after adjusting for both age and parasite positivity, the risk of malaria was reduced for individuals that were MBC positive for MSP-2 (3D7) (HR 0.22, 95% CI 0.05-0.98, *p*=0.047), and MSP-3 (HR 0.19, 95% CI 0.05-0.63, *p*=0.007).

**Table 3:** Memory B cell and antibody responses to *P. falciparum* antigens and risk of subsequent clinical malaria in malaria-exposed children (Junju).

Covariate	HR	95% CI	HRadj^a^ age	95% CI	HRadj^b^ parasites	95% CI	HRadj ^c^ age/parasite	95% CI
**MBC MSP-1_19_**	0.83	(0.37-1.87)	0.91	(0.40-2.11)	0.75	(0.33-1.71)	0.85	(0.37-1.96)
**MSP-2 (3D7)**	**0.18**	**(0.04-0.75)**	**0.17**	**(0.04-0.73)**	0.24	(0.05-1.03)	**0.22**	**(0.05-0.98)**
**MSP-2 (FC27)**	0.38	(0.09-1.58)	0.39	(0.09-1.71)	0.46	(0.11-1.91)	0.43	(0.1-1.09)
**MSP-3**	**0.17**	**(0.05-0.54)**	**0.15**	**(0.04-0.50)**	**0.21**	**(0.06-0.69)**	**0.19**	**(0.05-0.63)**
**AMA-1**	**0.41**	**(0.17-0.97)**	0.41	(0.16-1.00)	0.63	(0.25-1.61)	0.62	(0.23-1.62)
**CSP**	1.1	(0.34-3.61)	1.21	(0.37-3.98)	1.02	(0.31-3.33)	1.13	(0.34-3.71)
**Ab MSP-1_19_**	0.61	(0.33-1.13)	0.60	(0.32-1.11)	0.64	(0.34-1.19)	0.64	(0.34-1.19)
**MSP-2 (3D7)**	**0.36**	**(0.15-0.86)**	**0.36**	**(0.15-0.87)**	0.48	(0.19-1.16)	0.46	(0.18-1.13)
**MSP-2 (FC27)**	0.47	(0.14-1.50)	0.47	(0.15-1.50)	0.43	(0.13-1.38)	0.43	(0.13-1.38)
**MSP-3**	**0.36**	**(0.14-0.92)**	**0.36**	**(0.14-0.93)**	0.50	(0.19-1.32)	0.48	(0.17-1.29)
**AMA-1**	0.51	(0.25-1.01)	0.51	(0.24-1.05)	0.66	(0.32-1.37)	0.65	(0.31-1.37)
**CSP**	0.79	(0.24-2.55)	0.78	(0.24-2.52)	0.79	(0.24-2.54)	0.79	(0.24-2.55)
**MBC Breadth^d ^ 0**	1.00	ref	1.00	ref	1.0	ref	1.0	ref
**1**	0.58	(0.27-1.24)	0.56	(0.25-1.21)	0.57	(0.26-1.23)	0.55	(0.26-1.20)
**2**	0.47	(0.14-1.53)	0.47	(0.14-1.54)	0.55	(1.17-1.82)	0.57	(0.17-1.90)
**≥3**	**0.26**	**(0.08-0.86)**	**0.24**	**(0.07-0.84)**	0.39	(0.11-1.33)	0.34	(0.09-1.27)
**Ab Breadth** ^d^ 0	1.00	ref	1.0	ref	1.0	ref	1.0	ref
**1**	0.74	(0.44-1.23)	0.73	(0.39-1.35)	0.86	(0.46-1.06)	0.85	(0.45-1.59)
**2**	0.46	(0.17-1.19)	0.43	(0.16-1.17)	0.59	(0.22-1.58)	0.57	(0.21-1.54)
**≥3**	**0.20**	**(0.06-0.67)**	**0.19**	**(0.05-0.65)**	**0.26**	**(0.08-0.90)**	**0.24**	**(0.07-0.84)**

a Adjusted for age at sample collection.

b Adjusted for asymptomatic parasitaemia at sample collection.

c Adjusted for age at sample collection and for asymptomatic parasitaemia at sample collection.

d Breadth was defined as number of antigens (0-6) an individual had above threshold (≥20 spot-forming units for memory B cell responses or mean reactivity of malaria-naïve children + 2 SD for antibody responses). Values presented in bold represent a hazard ratio (HR) with 95% CI not crossing 1 and a p value below 0.05 therefore considered to be statistically significant.

In a separate univariate analysis within age groups, point estimates for MBC positive responses indicated reduced risk of malaria in older children (7-12 years), with significant associations for MSP-3 (HR 0.08 95% CI 0.01-0.64 *p*=0.017) (**Supplementary Table 1**). In contrast, the point estimates for MBC responses against CSP were increased but not significantly in younger children (**Supplementary Table 1**). For antibodies, the risk of clinical episode of malaria was significantly reduced in children aged 7-12 who were antibody positive for MSP-2 (3D7) (HR 0.37 95% CI 0.11-0.99 *p*=0.035) and MSP-3 (HR 0.33 95% CI 0.13-0.84 *p*=0.021) (**Supplementary Table 1**). A stratified analysis of the association between risk of malaria with MBC or antibody positivity in parasite positive versus negative children could not be tested due to small sample size.

**Figure 5:**
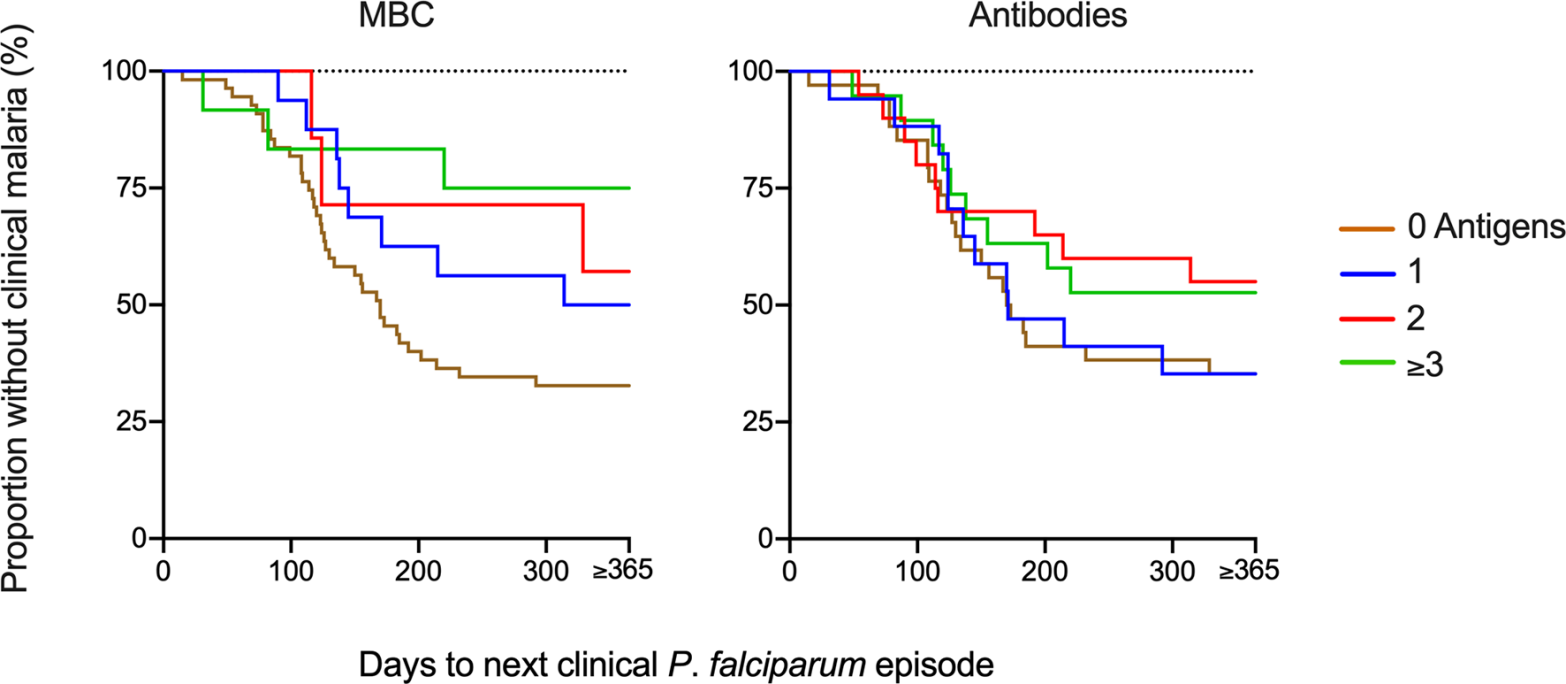
Risk of infection based on breadth of memory B-cell (MBC) and antibody responses at baseline. The breadth of the response was defined as the number of antigens against which a child had reactivity above threshold. The Kaplan-Meier curves present the proportion of malaria-exposed children (Junju) without clinical malaria and time to next malaria episode with regard to the breadth of MBC or antibody responses. The brown line represents a breadth of zero antigens; blue 1 antigen, red 2 antigens, and green line ≥3 antigens. Unadjusted and adjusted Cox regression analyses of the risk of malaria are presented in **Table 3** and **Supplementary Table 1**.

The breadth of antigen-specific MBC responses was associated with a reduced risk of malaria in children positive for 3 or more antigens in both the unadjusted analysis and after adjusting for age (**Table 3**, **Figure 5**). Similarly, when the analysis was split by age, there was a delay to the first clinical episode after baseline (HR 0.20, 95% CI 0.04-0.89, *p*=0.034) for children aged 7-12 and with a breadth of MBC responses exceeding 3 antigens (**Supplementary Table 1**). For antibodies, a breadth of ≥3 antigens were associated with a reduced risk of subsequent malaria in all children (HR 0.20, 95% CI 0.06-0.67, *p*=0.009) (**Table 3**) as well as children aged 7-12 years (HR 0.15, 95% CI 0.03-0.72, *p*=0.018) (**Supplementary Table 1**).

Lastly, we repeated the above analyses with the magnitudes of MBCs (number of antigen-specific SFU per 10^6^ PBMC) instead of MBC positivity, and the results were similar with the outcomes of the models based on MBC positivity. Similar to the Cox analysis based on MBC positivity (**Table 3**), magnitude of MBC responses to MSP-2 (3D7), MSP-3 and AMA-1 were associated with a reduced risk of subsequent malaria (**Supplementary Table 2**).

## Discussion

We assessed MBC and antibody responses specific for six well characterized *P. falciparum* antigens with risk of malaria in children living in an endemic area in Kenya. Our results indicate that levels for some of the merozoite MBC and antibody specificities were associated with a reduced risk of malaria. However, these associations were confounded by age and parasite positivity (at sampling) individually, and sometimes by both age and asymptomatic parasitemia. Importantly, a breadth of three or more antigens for either MBCs or antibodies remained protective even after controlling for age. Similarly, MBC positivity towards MSP-2 and MSP-3 remained protective after controlling for age and parasite positivity.

While the protective role of antibodies against *P. falciparum* antigens have been rigorously investigated (9, 43–45), the potential role of circulating MBCs in immunity remain understudied. Since MBCs need approximately 5-7 days to differentiate into antibody-secreting cells, their direct role in preventing new infections is unlikely. However, MBC could modulate the outcome of new infections by reducing their ability to induce malaria symptoms or reducing disease severity by rapidly differentiating into antibody secreting cells, upon reinfection. It is also likely that MBCs provide protection indirectly by frequently proliferating and differentiating into antibody secreting cells that keep replenishing *P. falciparum* specific bone marrow resident long-lived plasma cells that could maintain circulating antibodies at protective levels (46). That older children have both higher levels of MBCs and antibodies compared with younger ones, is consistent with our observation.

We found associations between MBC positivity for AMA-1, MSP-2 and MSP-3 and antibody positivity for MSP-2 (3D7) and MSP-3, as well as breadth of responses, with reduced risk of clinical *P. falciparum* malaria. However, some of these associations disappeared upon adjusting for age and/or asymptomatic parasitemia.

We also found that the number of antigen-specific MBCs varied between the tested antigens and was overall most pronounced for MSP-2 and MSP-3. In contrast, using the same FluoroSpot method including four antigens in adult Swedish travelers returning from the tropics with acute malaria, MBC responses were most pronounced for MSP-1_19_ and AMA-1 (30). This demonstrates that MBC responses can vary with study settings and illustrates the importance of including multiple antigens for the analysis within the same experiments. In our previous studies in travelers, it has also been shown that Africans with a history of exposure to *P. falciparum* did not respond to reinfection with higher frequencies of antigen-specific MBCs compared with first time-infected individuals, supporting our findings that a high parasite exposure is not necessarily correlated with increased number of parasite antigen-specific MBC in circulation (29, 30).

This study had some limitations. Despite the substantial number of children for this type of study, the statistical analyses including subgroup and multivariate analyses were restricted by relatively small sample sizes. For instance, the low number of children with asymptomatic parasitemia prevented us to properly perform statistical analysis of this group.

In addition, the children from Ngerenya village, included as a malaria-naïve controls, were relatively younger than the Junju children. Furthermore, malaria exposure among the control children was determined by passive surveillance and annual cross-sectional surveys, and although we define them as malaria-naïve, we cannot completely exclude the possibility of some limited exposure. Regarding the FluoroSpot methodology, reactivity to irrelevant tag antigens was low in general, and no cross-reactivity was detected between antigens, displaying the robustness of the assay.

In summary, we identified associations between levels and breadth of some antigen-specific MBC and antibody responses and reduced risk for malaria in children living in an endemic area. Antigen-specific MBC and antibody responses to merozoite antigens were associated with age and concurrent asymptomatic infections but not with the cumulative number of malaria episodes since birth. This would suggest that accumulated age, which would inevitably correlate with increased exposure, is more important for building the sufficient breadth for immunity compared to cumulative number of clinical episodes of malaria. Further studies will be needed to understand immunological memory in immunity to malaria.

## Data Availability Statement

The original contributions presented in the study are included in the article/**Supplementary Material**. Further inquiries can be directed to the corresponding authors.

## Ethics Statement

The studies involving human participants were reviewed and approved by The Kenyan Medical Research Institute National Ethics Committee (KEMRI SERU Protocol, No. 3149. Written informed consent to participate in this study was provided by the participants’ legal guardian/next of kin.

## Author Contributions

PJ designed and performed the study; analyzed and interpreted the data; and prepared the manuscript. DN assisted in sample collection, interpretation of the data, and data analysis. CS assisted in the design of the study, assisted with interpretation of data; supervised the study and revised the manuscript. LW assisted with statistical analysis of data. JMw, JMu, and EO assisted in sample collection and patient handling. NA assisted in interpretation of the data and revised the manuscript. PB assisted in the design of the study, assisted with interpretation of data and revised the manuscript. AF and FN designed the study, supervised the study implementation, analyzed the data, and revised the manuscript. All authors contributed to the article and approved the submitted version.

## Funding

This work was funded by The Swedish Foundation for Strategic Research (Industrial Doctoral grant ID14-0070), the Swedish Research Council (Development Research project grant 2018-04468) and Karolinska Institutet Travel Grant (2018-02525). FMN is supported by an MRC/UKRI African Research Leadership Award (MR/P020321/1), and Senior Fellowship from EDCTP (TMA2016SF).

## Conflict of Interest

PJ and NA are employed by Mabtech AB, Sweden.

The remaining authors declare that the research was conducted in the absence of any commercial or financial relationships that could be construed as a potential conflict of interest.

Several of the reagents and the FluoroSpot reader systems used in this study are produced by Mabtech. Mabtech has no influence on the content of this study or interpretation of results.

## Publisher’s Note

All claims expressed in this article are solely those of the authors and do not necessarily represent those of their affiliated organizations, or those of the publisher, the editors and the reviewers. Any product that may be evaluated in this article, or claim that may be made by its manufacturer, is not guaranteed or endorsed by the publisher.
