# Supplementary figures and images for: Plasmodium falciparum-Specific Memory B-Cell and Antibody Responses Are Associated With Immunity in Children Living in an Endemic Area of Kenya
Source: Front Immunol. 2022 Mar 9;13:799306. doi: 10.3389/fimmu.2022.799306 (PMC8959630; doi:10.3389/fimmu.2022.799306)

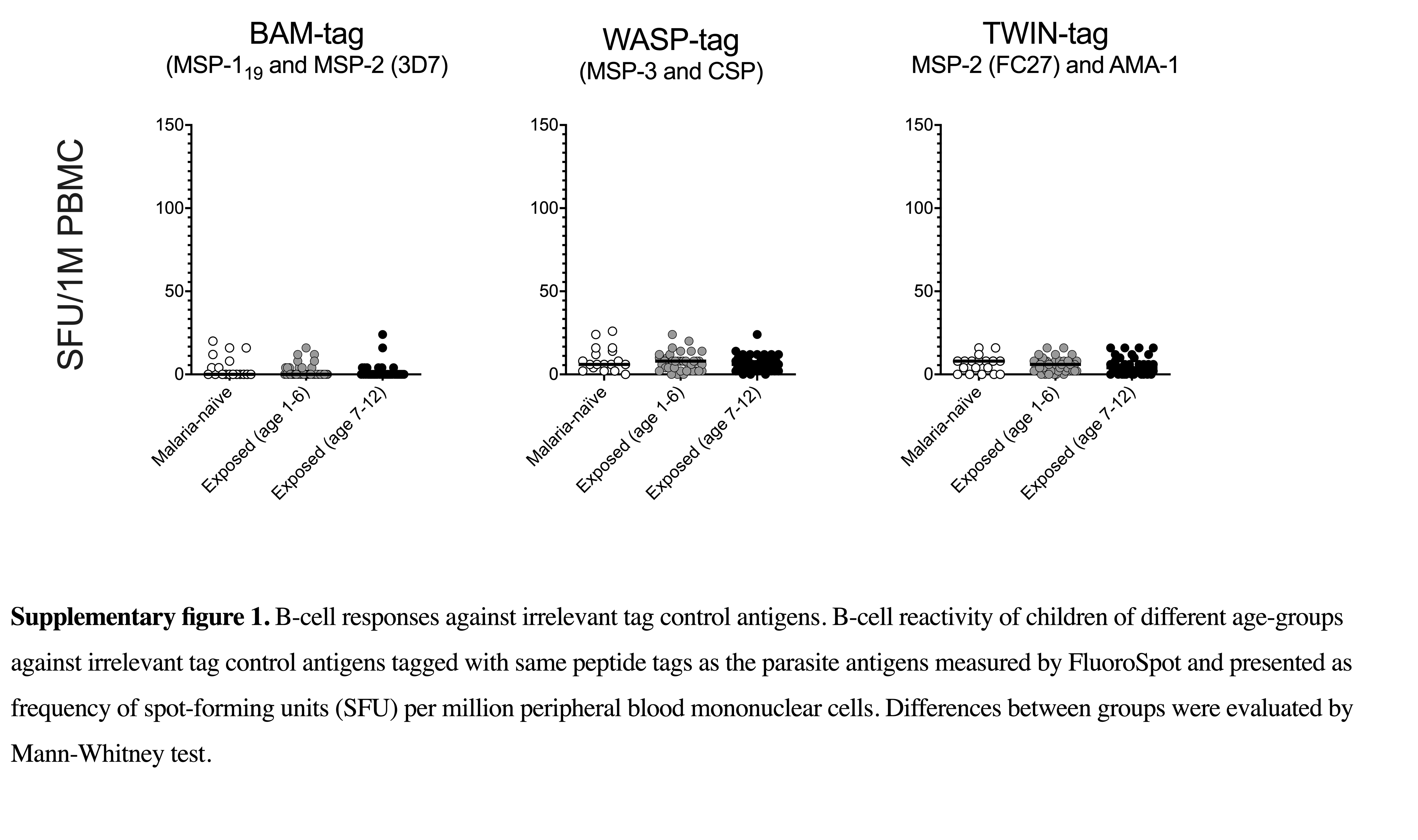

Supplement: Supplementary file 1 [file Image_1.tiff]

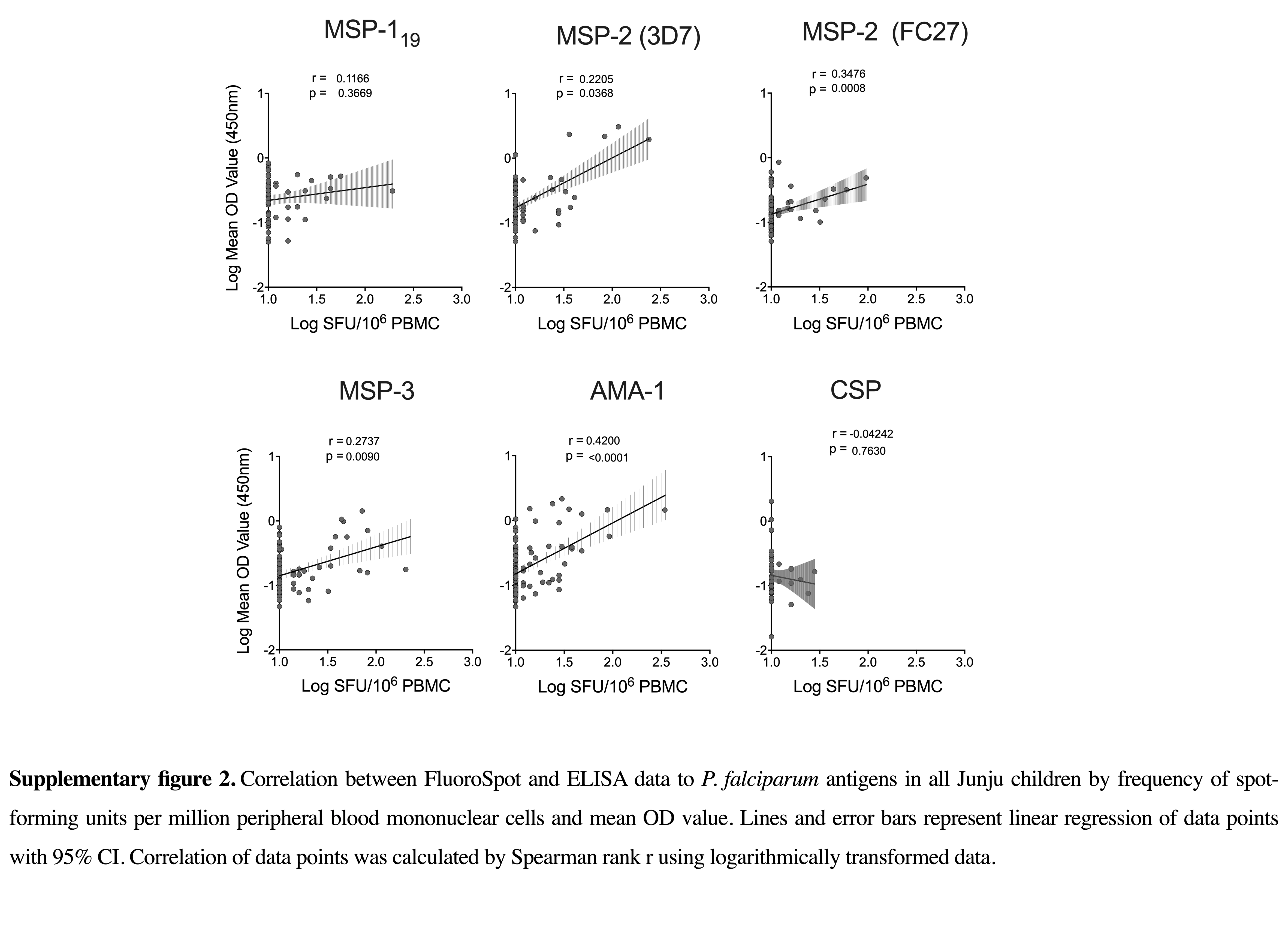

Supplement: Supplementary file 2 [file Image_2.tiff]

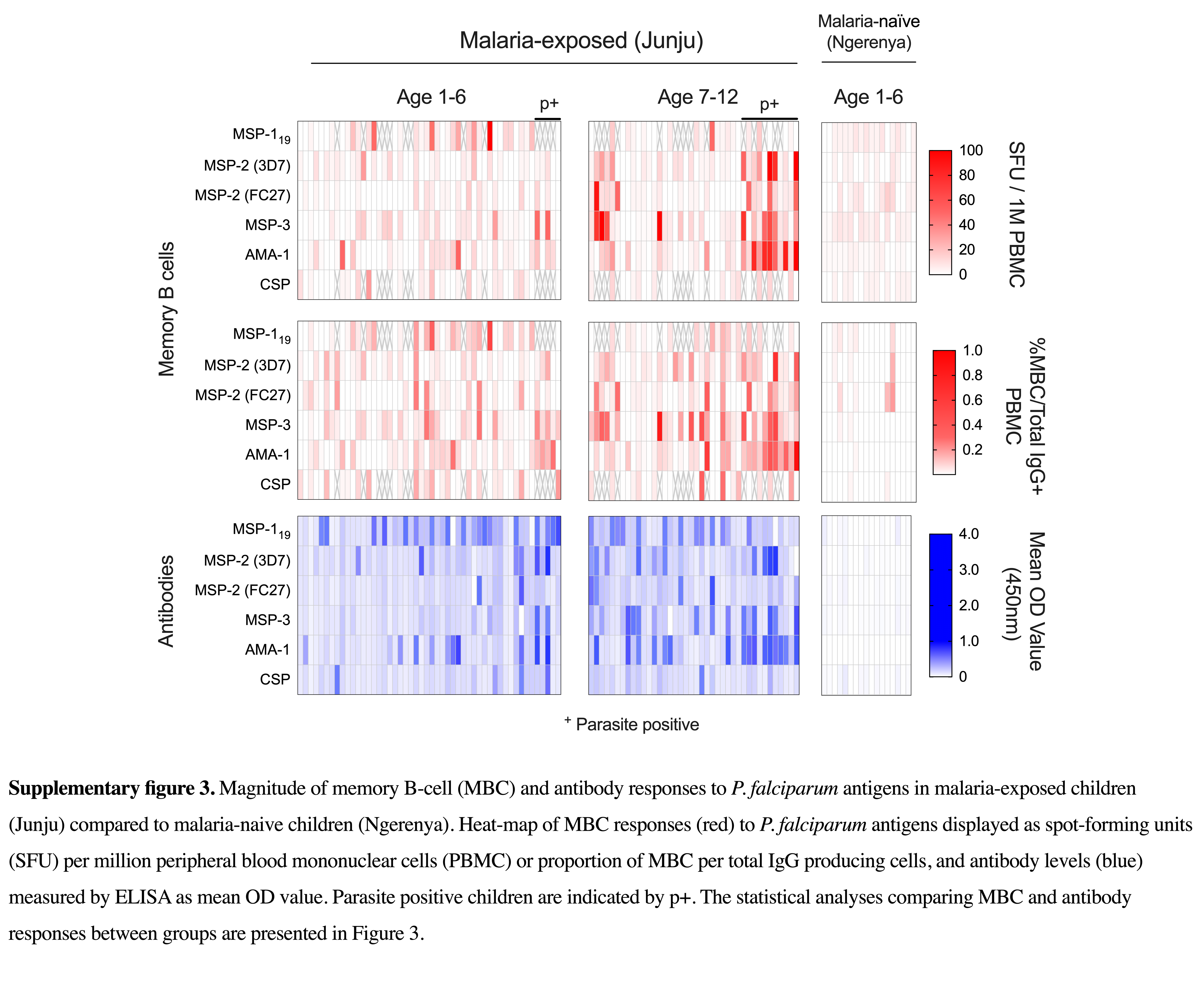

Supplement: Supplementary file 3 [file Image_3.tiff]
