# Supplementary material for: Plasmodium falciparum-Specific Memory B-Cell and Antibody Responses Are Associated With Immunity in Children Living in an Endemic Area of Kenya
Source: Front Immunol. 2022 Mar 9;13:799306. doi: 10.3389/fimmu.2022.799306 (PMC8959630; doi:10.3389/fimmu.2022.799306)
Supplement: Supplementary file 4 [file Table_1.docx]

**Supplementary table 1. Memory B cell and antibody responses to *P. falciparum* antigens and risk of subsequent clinical malaria in malaria-exposed children (Junju) of different age groups**

|  | **Age 1-6 years (n=50)** | | | | |  | **Age 7-12 years (n=40)** | | | | |  |  | |
| --- | --- | --- | --- | --- | --- | --- | --- | --- | --- | --- | --- | --- | --- | --- |
| **Covariate** | **HR** | **95% CI** |  | **HRadj^a^**  **age/parasite** | **95% CI** |  | **HR** | **95% CI** |  | **HRadj^a^**  **age/parasite** | **95% CI** |  |  |  |
| **MBC MSP-1_19_** | 0.66 | (0.25-1.75) |  | 0.76 | (0.28-2.08) |  | 1.43 | (0.32-6.31) |  | 1.39 | (0.31-6.23) |  |  |  |
| **MSP-2 (3D7)** | - | - |  | - | - |  | 0.28 | (0.06-1.20) |  | 0.56 | (0.09-3.33) |  |  |  |
| **MSP-2 (FC27)** | 3.57 | (0.46-27.5) |  | 3.83 | (0.48-30.0) |  | 0.22 | (0.03-1.64) |  | 0.33 | (0.04-2.75) |  |  |  |
| **MSP-3** | 0.34 | (0.08-1.44) |  | 0.41 | (0.09-1.74) |  | **0.08** | **(0.01-0.64)** |  | **0.11** | **(0.12-0.93)** |  |  |  |
| **AMA-1** | 0.71 | (0.21-2.34) |  | 0.95 | (0.27-3.29) |  | 0.30 | (0.06-1.03) |  | 0.51 | (0.12-2.29) |  |  |  |
| **CSP** | 1.14 | (0.34-3.82) |  | 0.94 | (0.28-3.18) |  | - | - |  | - | - |  |  |  |
| **Ab MSP-1_19_** | 0.65 | (0.34-1.22) |  | 0.83 | (0.44-1.57) |  | 0.62 | (0.22-1.69) |  | 0.6 | (0.21-1.67) |  |  |  |
| **MSP-2 (3D7)** | 0.59 | (0.21-1.67) |  | 2.49 | (0.63-9.80) |  | **0.33** | **(0.11-0.99)** |  | 0.38 | (0.12-1.16) |  |  |  |
| **MSP-2 (FC27)** | 0.5 | (0.15-1.65) |  | 1.15 | (0.34-3.90) |  | 0.35 | (0.05-2.64) |  | 0.31 | (0.04-2.37) |  |  |  |
| **MSP-3** | 0.45 | (0.11-1.87) |  | 2.13 | (0.45-9.90) |  | 0.36 | (0.12-1.08) |  | 0.47 | (0.15-1.42) |  |  |  |
| **AMA-1** | 0.79 | (0.35-1.79) |  | 1.24 | (0.53-2.87) |  | **0.27** | **(0.09-0.79)** |  | 0.36 | (0.11-1.16) |  |  |  |
| **CSP** | 0.18 | (0.02-1.34) |  | 0.34 | (0.40-2.70) |  | 1.69 | (0.37-6.91) |  | 1.45 | (0.33-6.41) |  |  |  |
| **MBC Breadth^b^ 0** | 1.00 | ref |  | 1.00 | ref |  | 1.00 | ref |  | 1.00 | ref |  |  |  |
| **1** | 0.90 | (0.34-2.38) |  | 0.78 | (0.29-2.09) |  | 0.35 | (0.10-1.23) |  | 0.39 | (0.11-1.39) |  |  |  |
| **2** | 0.63 | (0.19-2.12) |  | 0.70 | (0.21-2.37) |  | - | - |  | - | - |  |  |  |
| **≥3** | 0.51 | (0.07-3.82) |  | 0.81 | (0.10-6.24) |  | **0.20** | **(0.04-0.89)** |  | 0.31 | (0.05-2.09) |  |  |  |
| **Ab Breadth^b^ 0** | 1.00 | ref |  | 1.00 | ref |  | 1.00 | ref |  | 1.00 | ref |  |  |  |
| **1** | 0.74 | (0.38-1.43) |  | 1.04 | (0.53-2.02) |  | 0.74 | (0.27-2.01) |  | 0.57 | (0.19-1.68) |  |  |  |
| **2** | 0.55 | (0.19-1.63) |  | 0.64 | (0.22-1.91) |  | 0.26 | (0.05-1.24) |  | 0.39 | (0.06-1.38) |  |  |  |
| **≥3** | 0.37 | (0.08-1.60) |  | 1.85 | (0.39-8.72) |  | **0.15** | **(0.03-0.72)** |  | 0.24 | (0.04-1.00) |  |  |  |

Cox-regression analysis based on positivity. For memory B cell and for antibody responses, positivity was set to ≥20 of spot-forming units per million peripheral blood mononuclear cells or mean OD value of all malaria-naïve children plus 2 standard deviations (SD) respectively. Values with “-“ represent an analysis that could not be performed due to insufficient amount of data.

^a^ Adjusted for age and asymptomatic parasitaemia at sample collection^.^

^b^ Breadth was defined as number of antigens (0-6) an individual had above threshold (≥20 spot-forming units for memory B cell responses or mean reactivity of malaria-naïve children + 2 SD for antibody responses)
