# Supplementary material for: Plasmodium falciparum-Specific Memory B-Cell and Antibody Responses Are Associated With Immunity in Children Living in an Endemic Area of Kenya
Source: Front Immunol. 2022 Mar 9;13:799306. doi: 10.3389/fimmu.2022.799306 (PMC8959630; doi:10.3389/fimmu.2022.799306)
Supplement: Supplementary file 5 [file Table_2.docx]

**Supplementary table 2. Memory B cell and antibody responses to *P. falciparum* antigens and risk of subsequent clinical malaria in malaria-exposed children (Junju)**

|  |  |  |  | | | | | | | | | | |  | |  |  |
| --- | --- | --- | --- | --- | --- | --- | --- | --- | --- | --- | --- | --- | --- | --- | --- | --- | --- |
| **Covariate** | **HR** | | | **95% CI** |  | **HRadj^a^**  **age** | **95% CI** |  | **HRadj^b^ parasites** | **95% CI** | **HRadj ^c^**  **age/parasite** | **95% CI** |  | |  |  |  |
| **MBC MSP-1_19_** | 1.00 | | | (0.99-1.01) |  | 1.00 | (0.99-1.01) |  | 1.0 | (0.98-1.01) | 1.0 | (0.99-1.01) |  | |  |  |  |
| **MSP-2 (3D7)** | **0.96** | | | **(0.92-0.99)** |  | **0.95** | **(0.92-0.99)** |  | **0.96** | **(0.92-0.99)** | **0.96** | **(0.92-0.99)** |  | |  |  |  |
| **MSP-2 (FC27)** | 0.98 | | | (0.95-1.00) |  | 0.97 | (0.94-1.01) |  | 0.98 | (0.96-1.01) | 0.98 | (0.95-1.01) |  | |  |  |  |
| **MSP-3** | **0.98** | | | **(0.96-0.99)** |  | **0.98** | **(0.96-0.99)** |  | 0.99 | (0.97-1.00) | 0.99 | (0.97-1.00) |  | |  |  |  |
| **AMA-1** | **0.97** | | | **(0.95-0.99)** |  | **0.97** | **(0.95-0.99)** |  | 0.98 | (0.95-1.01) | 0.98 | (0.96-1.01) |  | |  |  |  |
| **CSP** | 1.02 | | | (0.98-1.06) |  | 1.06 | (0.98-1.14) |  | 1.02 | (0.98-1.06) | 1.03 | (0.99-1.07) |  | |  |  |  |

Cox-regression analysis based on frequency of spot-forming units per million peripheral blood mononuclear cells.

^a^ Adjusted for age at sample collection

^b^ Adjusted for asymptomatic parasitaemia at sample collection

^c^ Adjusted for age at sample collection and for asymptomatic parasitaemia at sample collection
